# Effects of USP Warm-Mix Modifier on Rheological Properties of PG76-22 Asphalt Binder and Performance of Modified Mixture

**DOI:** 10.3390/ma19122470

**Published:** 2026-06-09

**Authors:** Liusheng Hu, Xiyuan Shen, Zheng Wang, Ji Ma, Weiguang Zhang

**Affiliations:** 1 Guangde City Highway Development Center, Guangde 242200, China; uchan6514px@126.com; 2 School of Transportation, Southeast University, Nanjing 211189, China; wgzhang@seu.edu.cn; 3 Anhui Jiangong Road and Bridge Construction Group Co., Ltd., Hefei 239299, China; huqb416@163.com (Z.W.); chezhishi48411707@163.com (J.M.)

**Keywords:** USP modifier, asphalt binder, microscopic mechanism, rheological properties, pavement performance

## Abstract

The USP modifier is an environmentally friendly warm-mix asphalt additive that can reduce asphalt viscosity and contribute to energy savings and emission mitigation. In this study, the PG76-22 asphalt binder was used as the control material, and a USP-modified PG76-22 asphalt binder was prepared. Microscopic characterization tests, asphalt binder performance tests, and asphalt mixture performance tests were conducted to investigate the effects of the USP modifier on the PG76-22 asphalt binder and its mixtures. The FM observations showed that the USP modifier was relatively uniformly dispersed in the binder without obvious large-scale agglomeration, while the FTIR results showed no new major characteristic absorption peaks after USP modification. These results suggest that no evident chemical reaction was detected under the adopted test conditions. At the binder level, the USP modifier improved the low-temperature ductility of the PG76-22 asphalt binder but reduced its high-temperature deformation resistance, as indicated by a lower rutting factor, increased non-recoverable deformation under high stress, and enhanced stress sensitivity. The LAS results further showed that the fatigue life of the USP-modified asphalt binder was lower than that of the PG76-22 asphalt binder. At the mixture level, USP modification increased the dynamic stability, residual stability, and tensile strength ratio by 6.2%, 5%, and 3%, respectively, and resulted in longer four-point bending fatigue life at the tested strain levels. These results indicate limited improvements in the measured mixture-level performance under the present laboratory conditions. However, the reduced binder-level rutting resistance and LAS fatigue life suggest that USP modification exhibits different effects at the binder and mixture levels, and the mixture-level results should not be directly extrapolated from binder-level rheological performance alone.

## 1. Introduction

By the end of 2024, the total mileage of highways in China had exceeded 5.49 million km, among which expressways accounted for more than 190,000 km. Asphalt pavements account for more than 90% of high-grade highways [1,2]. With the continuous increase in traffic loads and the increasing complexity of service environments, higher requirements have been placed on the high-temperature stability, low-temperature cracking resistance, and fatigue resistance of asphalt materials used in high-grade highways [3]. The PG76-22 asphalt binder, as a typical performance-graded modified asphalt binder, has a high-temperature grade of 76 °C and a low-temperature grade of −22 °C. It exhibits favorable high-temperature deformation resistance and certain low-temperature adaptability and has been widely used in high-grade highways and heavy-duty traffic sections [4]. However, the PG76-22 asphalt binder is still commonly used in hot-mix asphalt systems, which generally require relatively high mixing and compaction temperatures to ensure aggregate coating and mixture workability [5]. These high-temperature processes increase energy consumption, accelerate asphalt aging, and generate carbon dioxide, asphalt fumes, and other harmful emissions, posing potential risks to the environment and construction workers [6,7].

To solve these problems, warm-mix asphalt (WMA) technology has been gradually applied in road engineering. This technology enables modified asphalt mixtures, including those prepared with the PG76-22 asphalt binder, to be mixed and compacted at lower temperatures by reducing asphalt viscosity, improving the coating condition between asphalt binder and aggregates, or enhancing mixture workability. In general, warm-mix asphalt technology can reduce the construction temperature by 20–30 °C while maintaining a pavement performance comparable to that of conventional hot-mix modified asphalt mixtures [8]. Therefore, introducing warm-mix modification technology into high-performance modified asphalt binders such as PG76-22 is of great significance for reducing construction temperature, decreasing pollutant emissions, mitigating asphalt thermal aging, and improving the sustainability of pavement construction [9].

Different WMA technologies achieve temperature reduction through different mechanisms. Foaming-based WMA temporarily increases the volume of the asphalt binder and improves coating workability, but its effectiveness is closely related to moisture control and foaming stability [10]. Organic viscosity-reducing additives can lower binder viscosity at construction temperatures; however, some additives may crystallize or increase binder stiffness after cooling, which may affect low-temperature cracking or fatigue behavior [11]. Chemical or surfactant-based additives mainly improve asphalt–aggregate coating and interfacial workability, but their effects may depend on aggregate type, binder chemistry, and aging conditions [12]. Therefore, the performance of WMA systems is not controlled only by viscosity reduction, but also by the interaction among modifier composition, binder rheology, aging behavior, and mixture structure [13].

In recent years, USP low-temperature modification technology has attracted increasing attention as an upgraded warm-mix technology. The USP modifier is prepared through chemical synthesis using waste tire rubber powder and waste plastics as the main raw materials, together with organic additives such as surfactants, catalysts, and polymer resins [14,15]. Compared with conventional warm-mix technologies, USP technology has several notable advantages. Studies have shown that its temperature-reduction range can reach 40 °C [16,17]. In terms of environmental benefits, the emissions of asphalt fumes and benzene-soluble substances can be reduced by more than 90%; the emissions of anthracene and naphthalene can be reduced by 89.3% and 86.6%, respectively [18]; sulfur dioxide and nitrogen oxide emissions can be reduced by more than 70%; and carbon dioxide emissions can be reduced by 56.4% [19]. In addition, this technology generally does not require major modifications to asphalt mixing plants, resulting in relatively low application costs and strong adaptability to different engineering scenarios [20].

However, the application of USP modifiers in high-performance polymer-modified asphalt binders such as PG76-22 still requires further investigation. The PG76-22 asphalt binder has relatively high stiffness and high-temperature deformation resistance, while the plasticizing, rubber/plastic-derived, resin, and surfactant-like components in USP may alter its phase morphology, FTIR-detectable functional-group characteristics, viscoelastic stiffness, creep-recovery behavior, and fatigue response [21]. Moreover, binder-scale rheological indices may not show the same trend as mixture-scale performance, because the rutting and fatigue resistance of asphalt mixtures are also influenced by asphalt content, aggregate structure, air void characteristics, asphalt–aggregate interaction, and deformation coordination [22]. This binder–mixture scale difference is particularly important for WMA systems, where improved workability and reduced construction temperature may benefit mixture performance, while the corresponding binder may exhibit reduced stiffness or altered stress sensitivity.

Previous studies on USP-modified asphalt materials have mainly focused on temperature reduction, viscosity reduction, emission mitigation, and selected engineering properties [23,24,25]. However, the application of the USP modifier in the PG76-22 asphalt binder still involves the following unresolved issues: (1) The influence of USP modification on the morphology, functional-group characteristics, and rheological properties of the PG76-22 asphalt binder remains insufficiently understood. (2) The effects of USP modification on PG76-22 asphalt mixture performance, particularly the difference between binder-scale and mixture-scale responses, have not been adequately investigated.

To address these issues, this study uses the PG76-22 asphalt binder as the control material and prepares a USP-modified PG76-22 asphalt binder. Fluorescence microscopy, Fourier transform infrared spectroscopy, and differential scanning calorimetry are first employed to characterize the microscopic morphology, chemical functional groups, and thermal behavior of the modified binder, thereby providing insight into the interaction between the USP modifier and PG76-22 asphalt binder. Subsequently, conventional asphalt binder tests, performance grading tests, multiple stress creep-recovery tests, linear amplitude sweep tests, temperature sweep tests, long-term creep tests, and asphalt mixture performance tests are conducted to systematically evaluate the effects of the USP modifier on the multi-scale performance of PG76-22 asphalt systems at both the binder and mixture levels. The findings are expected to provide a reference for the engineering application of USP low-temperature modified asphalt materials and the development of sustainable pavement materials.

## 2. Materials and Methods

### 2.1. Raw Materials

**Figure 1:**
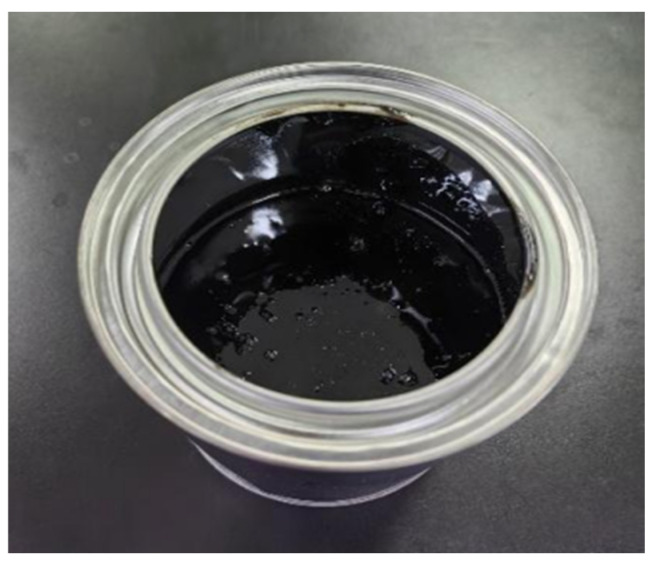
USP warm-mix modifier.

The raw materials used in this study mainly included the PG76-22 asphalt binder, USP modifier, coarse aggregate, fine aggregate, and mineral filler. The PG76-22 asphalt binder was provided by Jiangsu Yihu Asphalt Materials Co., Ltd. (Zhenjiang, China). Its basic properties were tested in the laboratory according to JTG E20-2011 [26], and the results are listed in Table 1. The USP modifier, supplied by Zhongyou Luzhixing New Materials Co., Ltd. (Zhengzhou, China), was prepared from waste tire rubber powder and waste plastic components through a synthetic modification process, with the addition of polymer resin, surfactants, catalysts/activating agents, and plasticizing or viscosity-reducing organic additives. At room temperature, it appears as a black or purple-black paste with no obvious odor and exhibits good storage stability and construction adaptability, as shown in Figure 1. The technical properties of the USP modifier are listed in Table 2.

The aggregates used for the asphalt mixtures were limestone aggregates, including coarse aggregate, fine aggregate, and filler. Their basic properties were tested according to JTG E42-2005 [27], and the results are presented in Table 3, Table 4 and Table 5.

### 2.2. Preparation of Modified Binders and Mixtures

The USP-modified PG76-22 asphalt binder was prepared using the melt blending method. First, the PG76-22 asphalt binder was heated to a flowable state and maintained under constant-temperature conditions to ensure good fluidity. The USP modifier and PG76-22 asphalt binder were then weighed at a mass ratio of 4.5:95.5 according to the technical recommendation provided by Zhongyou Luzhixing New Materials Co., Ltd. (Zhengzhou, China), and its suggested dosage range for engineering applications. The USP modifier was slowly added to the PG76-22 asphalt binder and initially stirred with a glass rod to obtain a preliminary blend. The blended asphalt binder was then subjected to high-speed shearing. Specifically, the mixture was first stirred at 1000 r/min for 10 min to promote preliminary dispersion of the USP modifier in the asphalt binder. The shearing speed was then increased to 5000 r/min and maintained for 30 min to enhance the dispersion of the USP modifier. Finally, the mixture was further stirred at 1000 r/min for 10 min to obtain a more homogeneous modified binder. After cooling, USP-modified PG76-22 asphalt binder with a USP content of 4.5% was obtained. To ensure consistency in the comparative tests, both the PG76-22 asphalt binder and USP-modified PG76-22 asphalt binder were tested under the same conditions in the subsequent microscopic characterization, conventional property tests, and rheological tests.

**Figure 2:**
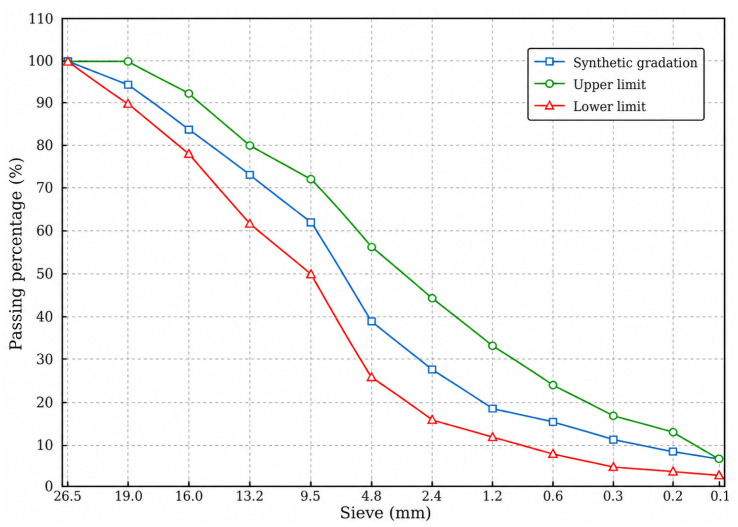
Gradation curve of AC-20 mixture.

According to the engineering requirements, AC-20 gradation was adopted for both the PG76-22 asphalt mixture and the USP-modified PG76-22 asphalt mixture [28], with the gradation curve and sieve passing percentages shown in Figure 2 and Table 6, respectively. Based on the mixture design results, the optimum asphalt–aggregate ratio was 4.2% for the USP-modified PG76-22 asphalt mixture and 4.7% for the PG76-22 asphalt mixture. During mixture preparation, the aggregates and mineral filler were first heated to the required temperature, and the PG76-22 asphalt binder or USP-modified PG76-22 asphalt binder was heated to a flowable state. The heated aggregates were then placed in the mixing pot and dry-mixed to ensure uniform temperature distribution. Subsequently, the corresponding asphalt binder was added for wet mixing until the aggregate surfaces were fully coated. Finally, the mineral filler was added and mixed until a homogeneous asphalt mixture was obtained. After mixing, rutting slabs, Marshall specimens, and indirect tensile specimens were prepared according to the requirements of different tests and cured under specified conditions for subsequent pavement performance evaluation.

### 2.3. Microscopic Characterization Tests

#### 2.3.1. Fluorescence Microscopy (FM) Test

The microscopic phase morphology of the PG76-22 asphalt binder and the USP-modified PG76-22 asphalt binder was examined using a BX53 fluorescence microscope (Olympus Corporation, Tokyo, Japan). For sample preparation, the asphalt binder was first heated to a flowable state (160 ± 5 °C). A small amount of the molten binder was then placed on a clean glass slide and covered with a cover slip. The specimen was briefly heated to promote uniform spreading and remove entrapped air bubbles and was subsequently cooled to room temperature before observation. The fluorescence phase morphology, particle dispersion, and possible agglomeration of the two binders were compared to evaluate the dispersion uniformity and compatibility of the USP modifier within the PG76-22 asphalt binder.

#### 2.3.2. Fourier Transform Infrared Spectroscopy Test (FTIR)

Fourier transform infrared spectroscopy was performed using a Nicolet iS50 FTIR spectrometer (Thermo Fisher Scientific, Waltham, MA, USA) to identify possible changes in the chemical functional groups of the PG76-22 asphalt binder after USP modification. The spectra of the PG76-22 asphalt binder and USP-modified PG76-22 asphalt binder were collected in attenuated total reflection mode over a wavenumber range of 4000–400 cm^−1^ at 25 °C, with 32 scans for each sample. The characteristic absorption peaks of the two binders were compared in terms of peak position, shape, and intensity to determine whether new functional groups were formed during modification and to clarify the interaction between the USP modifier and PG76-22 asphalt binder.

#### 2.3.3. Differential Scanning Calorimetry (DSC) Test

The thermal behavior of the USP modifier was characterized using a DSC 214 Polyma differential scanning calorimeter (NETZSCH-Gerätebau GmbH, Selb, Germany). Each sample was sealed in a crucible and tested under a nitrogen atmosphere at a flow rate of 20 mL/min. The temperature range was set from −50 °C to 150 °C, during which the heat flow response was recorded. The obtained DSC curves were used to identify the endothermic/exothermic transitions and to evaluate the effect of the USP modifier on the thermal response of the PG76-22 asphalt binder.

### 2.4. Asphalt Binder Performance Tests

#### 2.4.1. Penetration, Softening Point, and Ductility Tests

To evaluate the influence of the USP modifier on the conventional physical properties of the PG76-22 asphalt binder, penetration, softening point, and ductility tests were conducted in accordance with JTG E20-2011 [26]. Parallel specimens were prepared for each test, and the average value of the valid measurements was taken as the final result. These tests were used to characterize the binder consistency, high-temperature softening resistance, and low-temperature deformation capacity of the PG76-22 asphalt binder before and after USP modification.

#### 2.4.2. Performance Grading (PG) Test

**Figure 3:**
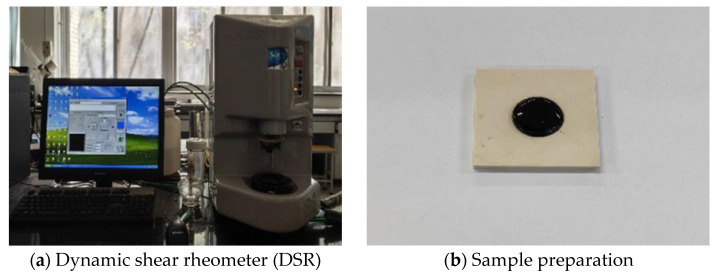
DSR and sample preparation.

To evaluate the influence of the USP modifier on the high-temperature and fatigue-related properties of the PG76-22 asphalt binder, tests were conducted using a DHR-2 dynamic shear rheometer (TA Instruments, New Castle, DE, USA) at 76 °C and a loading frequency of 10 rad/s, as shown in Figure 3. This test was used to compare the rheological response of the PG76-22 asphalt binder before and after USP modification at 76 °C PG temperature, rather than to determine the complete PG classification of the binders. The DSR test applies cyclic shear loading to characterize the viscoelastic response of an asphalt binder under traffic loading. Based on the test results, the failure temperature, rutting factor, and fatigue factor of the PG76-22 asphalt binder and USP-modified PG76-22 asphalt binder were compared and analyzed.

#### 2.4.3. Multiple Stress Creep-Recovery (MSCR) Test

To investigate the influence of the USP modifier on the high-temperature creep recovery and permanent deformation resistance of the PG76-22 asphalt binder, multiple stress creep-recovery (MSCR) tests were conducted in accordance with AASHTO T 350 [29]. The test temperature was set at 60 °C, which was selected as a representative high service temperature for comparing the stress-dependent creep-recovery behavior of the PG76-22 asphalt binder before and after USP modification. The test was performed under stress-controlled loading and recovery conditions.

For each specimen, 20 creep-recovery cycles were applied initially at a shear stress of 0.1 kPa, followed by 10 cycles at 3.2 kPa. Each cycle included 1 s of loading and 9 s of recovery. During the test, the strain responses of both the PG76-22 asphalt binder and USP-modified PG76-22 asphalt binder were recorded at the two stress levels. The average recovery rate, *R*, and non-recoverable creep compliance, $J_{nr}$, were calculated as follows:

(1) $$R=\frac{\varepsilon_{c}-\varepsilon_{r}}{\varepsilon_{c}-\varepsilon_{0}}$$

(2) $$J_{nr}=\frac{\varepsilon_{r}-\varepsilon_{0}}{\sigma }$$

where $\varepsilon_{0}$ is the initial strain at the beginning of each cycle; $\varepsilon_{c}$ is the peak strain at the end of the loading stage; $\varepsilon_{r}$ is the residual strain at the end of the recovery stage; and σ is the applied stress.

#### 2.4.4. Fatigue Performance Test

To evaluate the effect of the USP modifier on the fatigue damage resistance of the PG76-22 asphalt binder, a linear amplitude sweep (LAS) test was conducted using a DSR according to AASHTO TP 101 [30]. The test temperature was 25 °C. A linear amplitude sweep loading mode was applied at a fixed frequency of 10 Hz, and the shear strain increased gradually from 0% to 29%. The stress–strain responses of the USP-modified asphalt binder and PG76-22 asphalt binder during loading were recorded.

#### 2.4.5. Temperature Sweep Test

To investigate the effect of the USP modifier on the temperature-dependent viscoelastic behavior of the PG76-22 asphalt binder, temperature sweep tests were conducted using a DSR in accordance with AASHTO T 315 [31]. These tests spanned a temperature range from 0 °C to 80 °C and utilized strain-controlled loading with a strain amplitude of 1%. During the test, dynamic shear loading was applied to both the PG76-22 asphalt binder and the USP-modified PG76-22 asphalt binder. The storage modulus (*G*′), loss modulus (*G*″), and phase angle were recorded. Subsequently, the rutting factor was calculated based on the recorded rheological parameters.

#### 2.4.6. Long-Term Creep Test

To evaluate the effect of the USP modifier on the deformation resistance of the PG76-22 asphalt binder under long-term loading, long-term creep tests were conducted using a DSR. The test temperature was 60 °C. A constant shear stress of 0.1 kPa was applied to the two asphalt binders under stress-controlled mode for 1200 s, and the creep strain–time curves were recorded in real time.

### 2.5. Asphalt Mixture Performance Tests

#### 2.5.1. Rutting Test

According to JTG E20-2011 [26], rutting slab specimens of the PG76-22 asphalt mixture and USP-modified PG76-22 asphalt mixture were prepared with dimensions of 300 mm × 300 mm × 50 mm. The test temperature was set to 60 °C, the wheel pressure was 0.7 MPa, the loading speed was 42 passes/min, and the total loading time was 60 min. During the test, the rut depth was recorded as a function of loading time. The dynamic stability (*DS*) was calculated using the rut depths at 45 min and 60 min to evaluate the high-temperature rutting resistance of the two asphalt mixtures:

(3) $$DS=\frac{\left(t_{2}-t_{1}\right)\times N}{d_{2}-d_{1}}$$

where DS is the dynamic stability; $t_{1}$ and $t_{2}$ are 45 min and 60 min, respectively; $d_{1}$ and $d_{2}$ are the rut depths at 45 min and 60 min, respectively; and N is the wheel tracking speed.

#### 2.5.2. Moisture Stability Test

Moisture stability tests were conducted in accordance with JTG E20-2011 [26] using residual stability and the tensile strength ratio (TSR) as evaluation indices. For the residual stability test, Marshall specimens of the PG76-22 asphalt mixture and the USP-modified PG76-22 asphalt mixture were divided into a standard group and an immersed group. The standard specimens were conditioned in a 60 °C water bath for 30–40 min before Marshall stability testing, whereas the immersed specimens were soaked in a 60 °C water bath for 48 h before testing. The residual stability was then calculated to evaluate the strength retention of the mixtures after moisture conditioning:

(4) $${MS}_{0}=\frac{{MS}_{1}}{MS}\times 100\%$$

where ${MS}_{0}$ is the residual stability; MS is the Marshall stability under standard conditions; and ${MS}_{1}$ is the Marshall stability after 48 h immersion.

In the TSR test, the specimens were divided into an unconditioned group and a freeze–thaw-conditioned group. The conditioned group was subjected to vacuum saturation, low-temperature freezing, thawing in a 60 °C water bath, and conditioning in a 25 °C water bath before splitting strength testing. The tensile strength ratio was calculated by comparing the splitting tensile strengths before and after freeze–thaw conditioning:

(5) $$TSR=\frac{{RT}_{2}}{{RT}_{1}}\times 100\%$$

where TSR is the tensile strength ratio; ${RT}_{1}$ is the splitting tensile strength of the unconditioned group; and ${RT}_{2}$ is the splitting tensile strength of the freeze–thaw-conditioned group.

#### 2.5.3. Fatigue Performance of Asphalt Mixtures

Four-point bending fatigue tests were conducted in accordance with AASHTO T 321 [32] to evaluate the fatigue performance of the PG76-22 asphalt mixture and the USP-modified PG76-22 asphalt mixture. The tests were performed under strain-controlled loading at strain levels of 300, 400, and 500 με. The test temperature was 15 °C, and the loading frequency was 10 Hz. Fatigue life was defined as the number of load cycles at which the stiffness modulus decreased to 50% of its initial value. The fatigue lives of the two asphalt mixtures were then compared at different strain levels.

To ensure the reliability and repeatability of the experimental results, parallel specimens were prepared for all tests. For the conventional asphalt binder tests, rheological tests, rutting tests, and four-point bending fatigue tests, three valid replicate specimens or measurements were used for each test condition. For the moisture stability tests, parallel specimens were prepared for both the conditioned and unconditioned groups according to the corresponding standard test methods.

## 3. Results and Discussion

### 3.1. Microscopic Mechanism Analysis

#### 3.1.1. Fluorescence Microscopy (FM) Analysis

**Figure 4:**
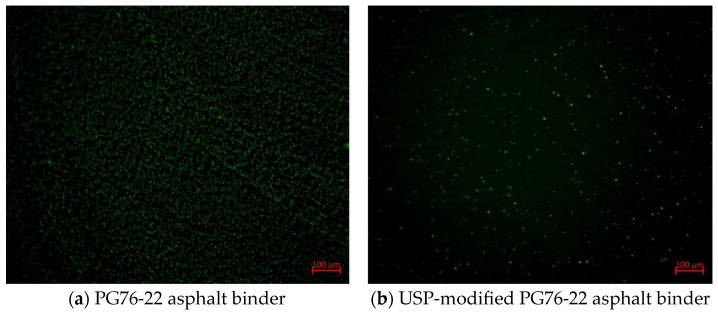
Fluorescence microscopy images of different asphalt binders.

Figure 4 shows the fluorescence microscopy images of the PG76-22 asphalt binder and USP-modified PG76-22 asphalt binder. As shown in Figure 4a, the PG76-22 asphalt binder exhibits a continuous and relatively uniform fluorescent phase under excitation, with no obvious large-scale agglomeration or phase separation. This indicates good compatibility between the modified components and the asphalt matrix. After the addition of the USP modifier, as shown in Figure 4b, a number of fluorescent dispersed particles appear in the asphalt binder system. These particles are mainly associated with the resin, rubber powder, and organic additives in the USP modifier, indicating that these components entered the PG76-22 asphalt binder and altered its original phase structure.

The fluorescence microscopy images were further analyzed using ImageJ software (version 1.53). The fluorescent dispersed phase was segmented by grayscale conversion and thresholding, and the average equivalent particle diameter, particle area fraction, number density, and dispersion coefficient were calculated. As shown in Table 7, no obvious discrete fluorescent particles were detected in the PG76-22 asphalt binder. After USP modification, dispersed fluorescent particles appeared in the asphalt matrix, with an average equivalent diameter of 6.08 μm, a particle area fraction of 0.20%, a number density of 57.66 particles·mm^−2^, and a dispersion coefficient of 0.45. These results indicate that the fluorescent particles were relatively fine and moderately uniformly distributed, with no obvious large-scale agglomeration in the selected field of view. Therefore, under the adopted preparation and observation conditions, the USP modifier was relatively well dispersed in the PG76-22 asphalt binder. However, further storage stability and phase-separation tests are still needed to quantitatively evaluate the long-term compatibility of the modified binder.

#### 3.1.2. Fourier Transform Infrared Spectroscopy (FITR) Analysis

**Figure 5:**
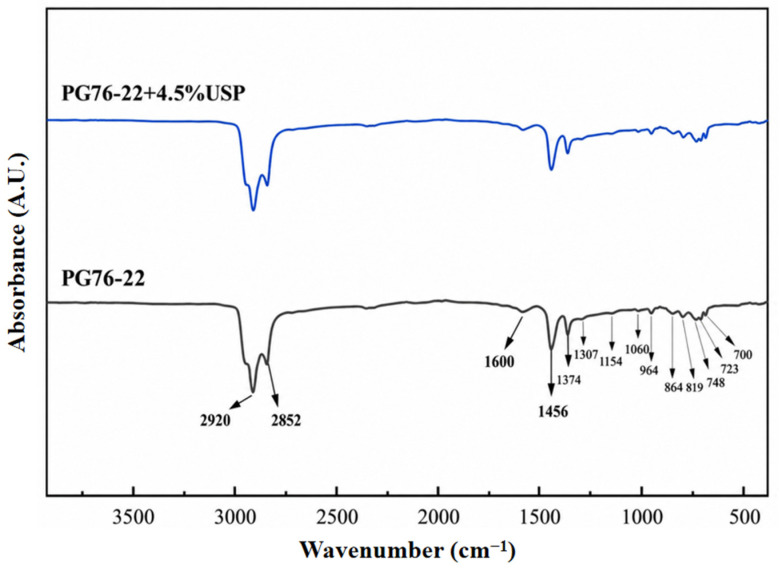
FTIR spectrums of different asphalts.

Figure 5 presents the FTIR spectra of the PG76-22 asphalt binder and USP-modified PG76-22 asphalt binder. Both binders show similar characteristic absorption peaks, with prominent bands near 2916, 2847, 1453, 1375, and 750 cm^−1^. No new absorption peaks appear in the functional group region of 4000–1300 cm^−1^ after USP modification. The peaks at approximately 2916 and 2847 cm^−1^ are attributed to the stretching vibrations of C-H bonds in methyl (-CH_3_) and methylene (-CH_2_-) groups, while those near 1453 and 1375 cm^−1^ correspond to C-H bending vibrations. The peak around 750 cm^−1^ is associated with out-of-plane C-H bending vibrations in aromatic rings. These results suggest that the USP modifier does not introduce new major functional groups into the PG76-22 asphalt binder.

After USP modification, slight changes in peak intensity were observed in the C-H stretching vibration region of 2800–3000 cm^−1^ and the C-H bending vibration region of 1350–1470 cm^−1^. These changes may be related to the incorporation of rubber, plastic, resin, and organic additive components from the USP modifier into the asphalt binder system [33].

To further quantify the FTIR results, the carbonyl index, sulfoxide index, and characteristic peak area ratios were calculated from the FTIR spectra. As shown in Table 8, the carbonyl index changed slightly from 0.026 for the PG76-22 asphalt binder to 0.029 for the USP-modified PG76-22 asphalt binder, while the sulfoxide index increased from 0.010 to 0.018. Both indices remained at relatively low levels. In addition, the A2920/A1456 and A1456/A1374 ratios changed from 1.53 and 1.97 to 2.83 and 2.05, respectively, indicating changes in the relative intensity of aliphatic C-H absorption bands. Overall, the FTIR results suggest that USP modification mainly affected the relative intensity of existing characteristic peaks, rather than producing distinct new absorption peaks.

#### 3.1.3. Differential Scanning Calorimetry (DSC) Analysis

**Figure 6:**
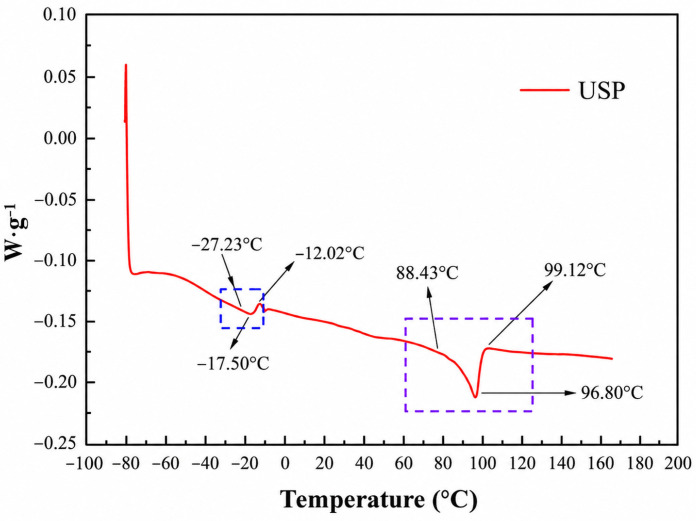
DSC curve of the USP warm-mix modifier. The blue dashed box highlights the low-temperature thermal transition region, and the purple dashed box highlights the main softening or melting transition region of the USP modifier.

Figure 6 shows the DSC curve of the USP modifier. During heating, a distinct downward endothermic peak is observed, indicating that the USP modifier undergoes an endothermic transition and gradually changes from a paste-like or semi-solid state to a flowable state. Based on the position of the endothermic peak, the melting or softening transition of the USP modifier mainly occurs within the range of 88–99 °C. This range is lower than the conventional mixing and compaction temperatures of asphalt mixtures, suggesting that the USP modifier can maintain better fluidity under practical construction conditions. This facilitates its dispersion in the PG76-22 asphalt binder and contributes to its viscosity-reducing effect.

The DSC results indicate that the USP modifier exhibits a clear thermal response during heating. The observed endothermic transition suggests that the modifier becomes more flowable within the tested temperature range, which may contribute to its warm-mix viscosity-reducing effect. Combined with the FM and FTIR results, the interaction between the USP modifier and PG76-22 asphalt binder appears to be dominated by physical blending and phase regulation under the present test conditions, rather than by the formation of new FTIR-detectable chemical bonds. Nevertheless, the specific contribution of each component in the USP modifier to the viscoelastic response and thermal behavior of the binder requires further verification through more detailed component-level analysis.

### 3.2. Analysis of Conventional Asphalt Binder Properties

#### 3.2.1. Penetration

**Figure 7:**
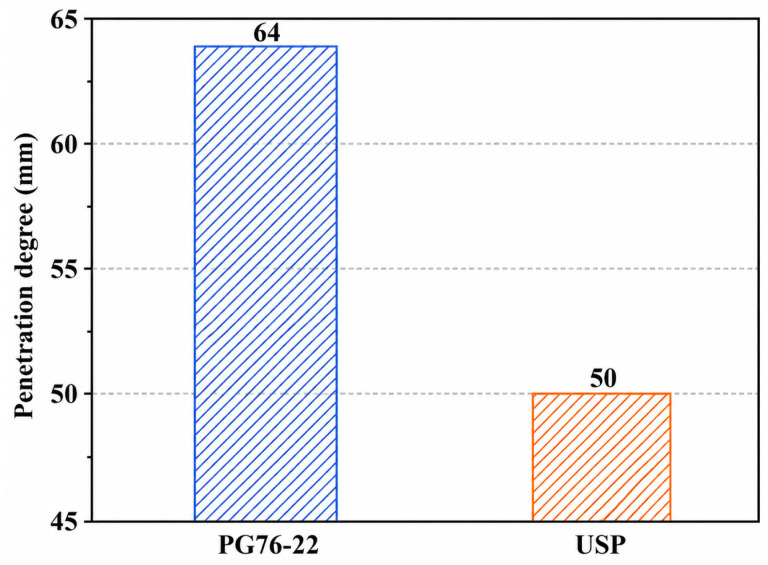
Effect of the USP modifier on the penetration of the PG76-22 asphalt binder.

Figure 7 compares the penetration values of the two asphalt binders. The penetration value decreases from 64 for the PG76-22 asphalt binder to 50 after USP modification, resulting in a reduction of 21.9%. This suggests that the USP modifier enhances the binder’s consistency at 25 °C and improves its resistance to deformation at room temperature.

#### 3.2.2. Softening Point

**Figure 8:**
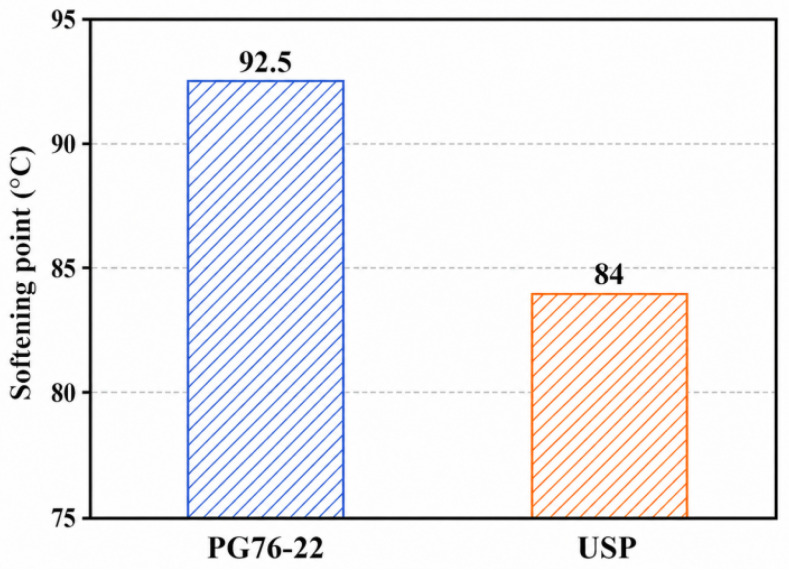
Effect of the USP modifier on the softening point of the PG76-22 asphalt binder.

Figure 8 compares the softening points of the two asphalt binders. The softening point of the PG76-22 asphalt binder is 92.5 °C, whereas that of the USP-modified PG76-22 asphalt binder decreases to 84 °C after adding the USP modifier, corresponding to a reduction of 9.2%. This indicates that the USP modifier reduces the resistance of the PG76-22 asphalt binder to flow deformation under heating conditions.

#### 3.2.3. Ductility

**Figure 9:**
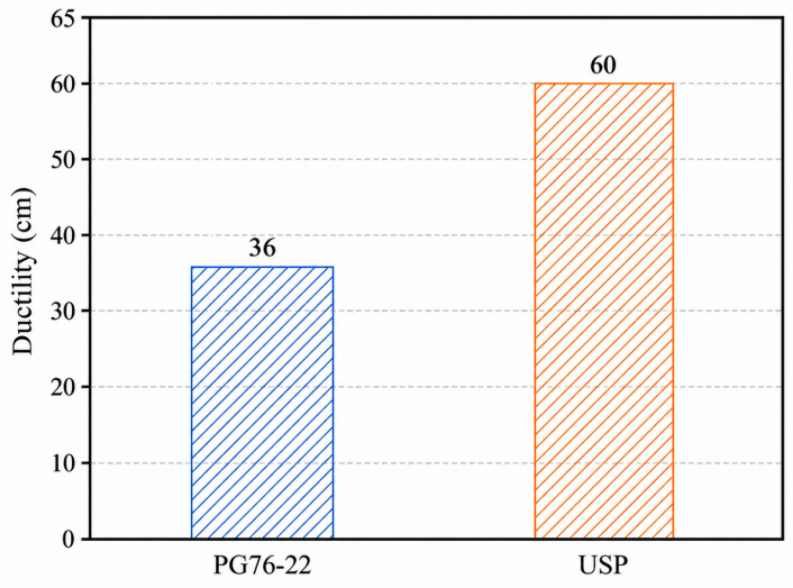
Effect of the USP modifier on the ductility of the PG76-22 asphalt binder.

Figure 9 compares the ductility of the two asphalt binders. After USP modification, the 5 °C ductility increases from 36 cm to 60 cm, corresponding to an increase of 66.7%. This indicates that the USP modifier significantly improves the low-temperature ductility and cracking resistance of the PG76-22 asphalt binder.

### 3.3. Rheological Properties of Asphalt Binder

#### 3.3.1. Analysis of Performance Grading (PG) Results

Table 9 presents the effect of the USP modifier on the performance grading indices of the PG76-22 asphalt binder. Compared with the original PG76-22 asphalt binder, USP modification reduces the failure temperature by approximately 2.5 °C, the rutting factor by 26.8%, and the fatigue factor by 27.8%. The decreases in failure temperature and rutting factor indicate that USP modification reduced the high-temperature deformation resistance of the PG76-22 asphalt binder at the binder scale. This result is consistent with the lower modulus response observed in the temperature sweep test, suggesting that the USP modifier reduced the stiffness and viscoelastic resistance of the binder system under high-temperature loading. The low-viscosity and plasticizing components in the USP modifier may contribute to the lower high-temperature stiffness, thereby leading to a reduction in rutting factor and failure temperature.

Comparison of the USP-modified PG76-22 asphalt binder before and after aging shows that the failure temperature changes only slightly, whereas the rutting factor and fatigue factor increase by 109% and 88.9%, respectively. This may be attributed to the volatilization or migration of light components and organic additives in the USP modifier during short-term aging, as well as the hardening of the asphalt binder system. These changes increase the complex modulus of the modified binder, thereby leading to higher rutting and fatigue factors.

#### 3.3.2. Analysis of Multiple Stress Creep-Recovery (MSCR) Results

**Figure 10:**
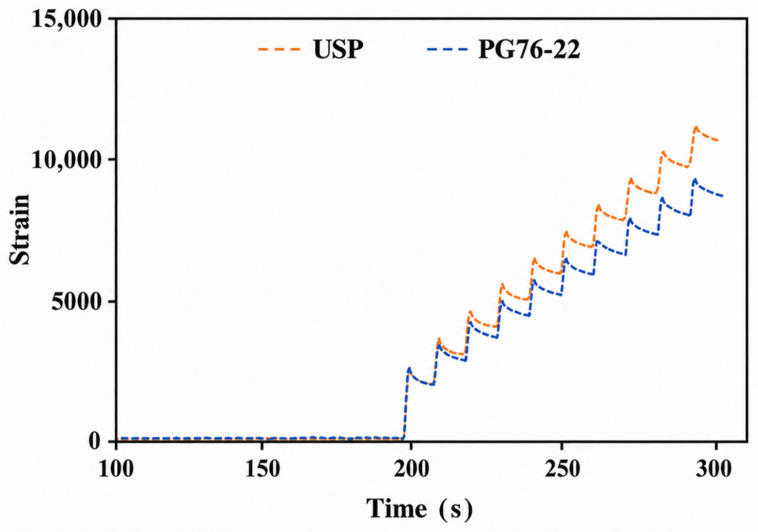
Effect of the USP modifier on the MSCR behavior of the PG76-22 asphalt binder.

Figure 10 shows the effect of the USP modifier on the MSCR behavior of the PG76-22 asphalt binder. As shown in the figure, the accumulated strain of the USP-modified asphalt binder is greater than that of the original PG76-22 asphalt binder as loading time increases. This indicates that the USP modifier reduces the resistance of the binder to permanent deformation under high-temperature loading.

**Figure 11:**
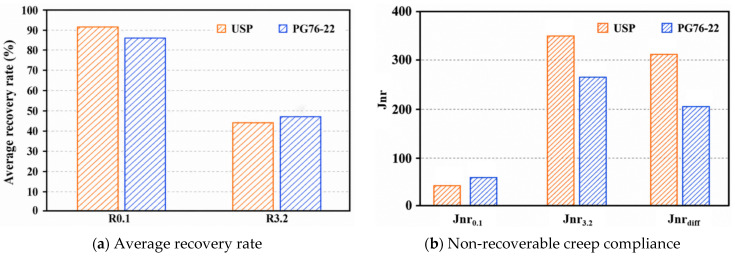
Effect of the USP modifier on average recovery rate and non-recoverable creep compliance of the PG76-22 asphalt binder.

Figure 11 demonstrates the impact of the USP modifier on the average recovery rate and non-recoverable creep compliance of the PG76-22 asphalt binder. Under low-stress conditions, the USP modification results in a 5% increase in the average recovery rate and a 20% decrease in non-recoverable creep compliance. Conversely, under high-stress conditions, the average recovery rate experiences a decrease of 3.3%, while non-recoverable creep compliance increases by 31.8%. These results indicate that the influence of the USP modifier on the creep-recovery behavior of the PG76-22 asphalt binder is strongly stress-dependent. At low stress, the structural constraint effect of the dispersed phase is more pronounced; at high stress, viscous flow caused by the plasticizing and viscosity-reducing effect dominates, resulting in reduced permanent deformation resistance. The addition of USP increases the $J_{nr,diff}$ value by 50%, indicating increased stress sensitivity, weakened deformation recovery capacity, and reduced rutting resistance.

The standard deviations of the recovery rate and $J_{nr}$ were relatively small, with CV values below 5%, indicating good repeatability of the MSCR results. The differences in *R*, $J_{nr}$, and $J_{nr,diff}$ between the PG76-22 asphalt binder and USP-modified PG76-22 asphalt binder were statistically significant (*p* < 0.05), confirming the stress-dependent effect of the USP modifier on creep-recovery behavior. The detailed statistical results are summarized in Section 3.5. However, MSCR tests were conducted only at 60 °C in this study. Therefore, the temperature dependence of stress sensitivity and rutting susceptibility should be further investigated in future work.

#### 3.3.3. Analysis of Fatigue Performance Results

**Figure 12:**
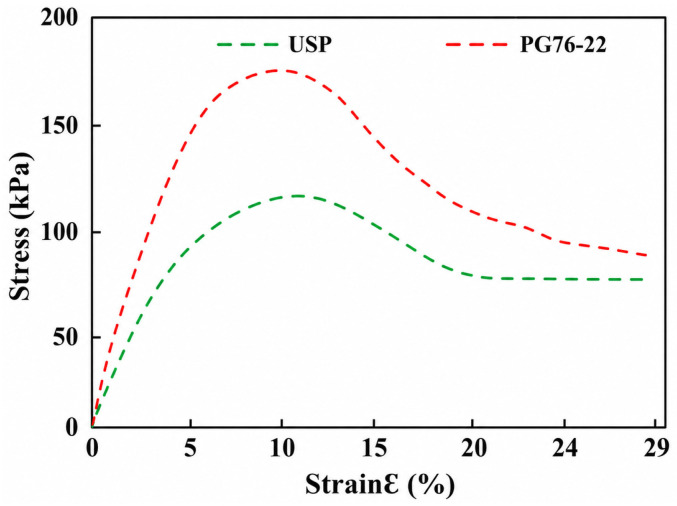
Effect of the USP modifier on the stress–strain response of the PG76-22 asphalt binder in the LAS test.

Figure 12 shows the stress–strain results from the LAS fatigue test. Both asphalt binders exhibit a stress response that first increases and then decreases with increasing shear strain. The strain levels corresponding to the peak stress are 10.81% and 10.52%, respectively, indicating that the USP modifier has little influence on the failure strain level of the PG76-22 asphalt binder. However, the peak stress of the USP-modified PG76-22 asphalt binder is significantly lower than that of the PG76-22 asphalt binder, with the peak stress of the PG76-22 asphalt binder being approximately 60% higher. This indicates that the USP modifier reduces the stress response and load-bearing capacity of the asphalt binder under cyclic shear loading. This behavior may be related to the plasticizing and viscosity-reducing components in the USP modifier, which could reduce binder stiffness and cohesive resistance. Since no new major FTIR absorption peaks were observed, the change was more likely associated with changes in physical blending, phase structure, and binder stiffness. Further chemical and microstructural analyses are needed to verify this interpretation.

To further evaluate the fatigue performance of the two asphalt binders, damage parameters were calculated from the LAS test data, as shown in Table 10. The A value of the USP-modified PG76-22 asphalt binder is 1.31 × 10^7^, which is lower than that of the PG76-22 asphalt binder (2.97 × 10^7^), whereas the B values of the two binders are similar. This indicates that the USP modifier mainly reduces the fatigue life capacity of the asphalt binder.

**Figure 13:**
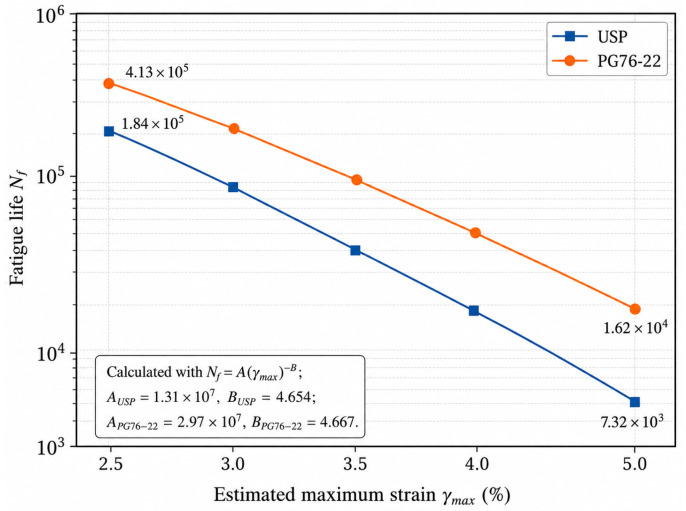
Estimated fatigue life.

Figure 13 shows the estimated fatigue life of the two binders. At strain levels of 2.5% and 5%, the fatigue life of the USP-modified PG76-22 asphalt binder is 53% and 51% lower, respectively, than that of the PG76-22 asphalt binder. Therefore, although the USP modifier reduces the stress level and initial stiffness of the asphalt binder, it does not improve fatigue life at the binder scale according to the LAS evaluation. This reduction in fatigue life may be related to the plasticizing and viscosity-reducing effect of the USP modifier, as well as the influence of resin components on binder cohesion.

However, the LAS test was conducted only on unaged binders to evaluate the direct effect of USP modification before aging. Therefore, the influence of RTFOT and PAV aging on binder-scale fatigue performance should be further investigated in future work.

#### 3.3.4. Analysis of Temperature Sweep Results

**Figure 14:**
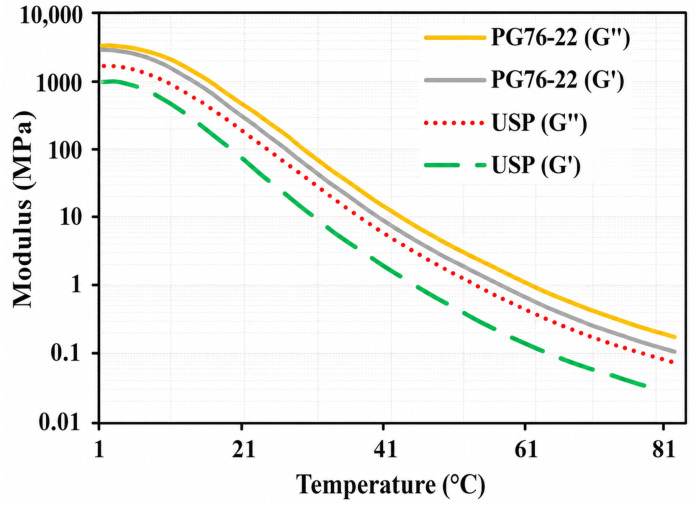
Effect of the USP modifier on storage modulus and loss modulus of the PG76-22 asphalt binder.

As shown in Figure 14, the storage modulus and loss modulus of both asphalt binders gradually decrease as temperature increases, indicating that the viscoelastic response of the binder weakens at higher temperatures. Compared with the PG76-22 asphalt binder, the USP-modified PG76-22 asphalt binder exhibits lower storage and loss moduli over the entire temperature range, suggesting that the USP modifier reduces binder stiffness and viscous resistance. This reduction is mainly attributed to the plasticizing and viscosity-reducing components in the USP modifier, which weaken intermolecular cohesion within the asphalt binder and lower the overall modulus level. In the medium-to- high-temperature range of 25–70 °C, the loss modulus of the PG76-22 asphalt binder is approximately 30–50% higher than that of the USP-modified binder, indicating a stronger viscous response. After USP modification, the lower loss modulus reflects the softening and viscosity-reducing effect of the modifier. However, the reduced modulus level may also weaken the binder-scale resistance to high-temperature deformation.

**Figure 15:**
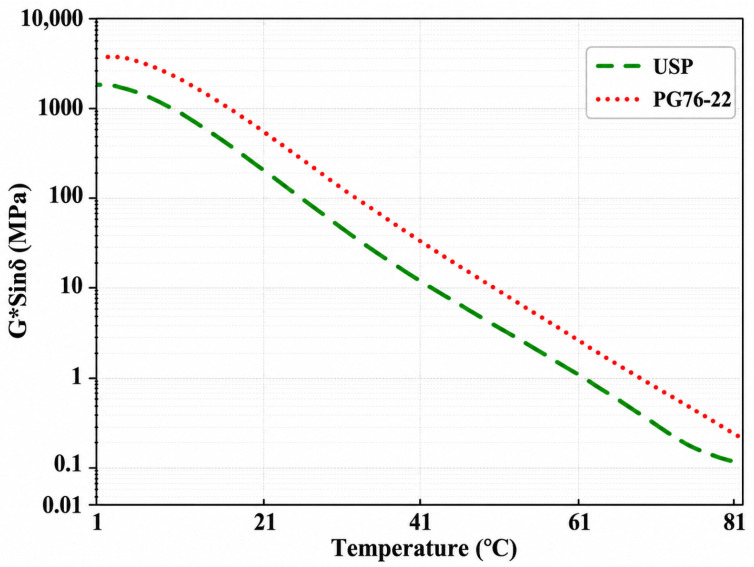
Effect of USP modifier on rutting factor of PG76-22 asphalt.

As shown in Figure 15, the rutting factors of both asphalt binders gradually decrease with increasing temperature, indicating a reduction in high-temperature deformation resistance. Within the range of 0–50 °C, the PG76-22 asphalt binder exhibits a significantly higher rutting factor than the USP-modified PG76-22 asphalt binder, suggesting greater viscoelastic stiffness and deformation resistance in this temperature range. After USP modification, the rutting factor of the PG76-22 asphalt binder decreases overall, indicating that the USP modifier weakens binder-scale rutting resistance. This is mainly attributed to the warm-mix viscosity-reducing effect of the USP modifier, as its plasticizing and viscosity-reducing components reduce the viscoelastic stiffness of the asphalt binder system. In the high-temperature range of 50–80 °C, the difference between the two binders gradually narrows, which may be related to the migration or volatilization of light components in the USP modifier and the structural regulation provided by resin, rubber powder, and other components.

#### 3.3.5. Analysis of Long-Term Creep Results

**Figure 16:**
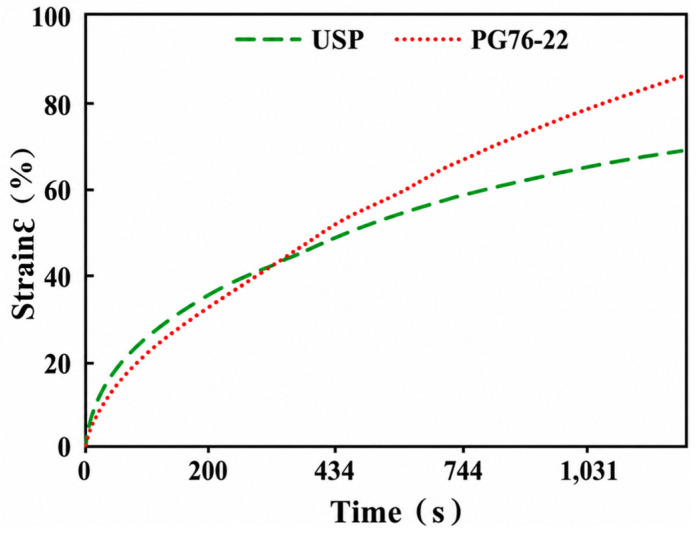
Effect of the USP modifier on long-term creep strain of the PG76-22 asphalt binder.

Figure 16 shows the effect of the USP modifier on the long-term creep strain of the PG76-22 asphalt binder. During the initial loading stage, the creep strain of the USP-modified binder is slightly higher than that of the original PG76-22 binder. As loading continues, the creep strain of the PG76-22 asphalt binder increases more rapidly, and the two curves intersect at approximately 300 s. After further loading, the creep strain of the PG76-22 asphalt binder becomes 16% higher than that of the USP-modified binder. This indicates that USP modification changed the time-dependent creep response of the PG76-22 asphalt binder. The lower creep strain in the later stage may be related to the combined effect of the dispersed modifier phase and the viscoelastic response of the binder system under sustained loading.

### 3.4. Analysis of Asphalt Mixtures Properties

#### 3.4.1. High-Temperature Stability Analysis

**Figure 17:**
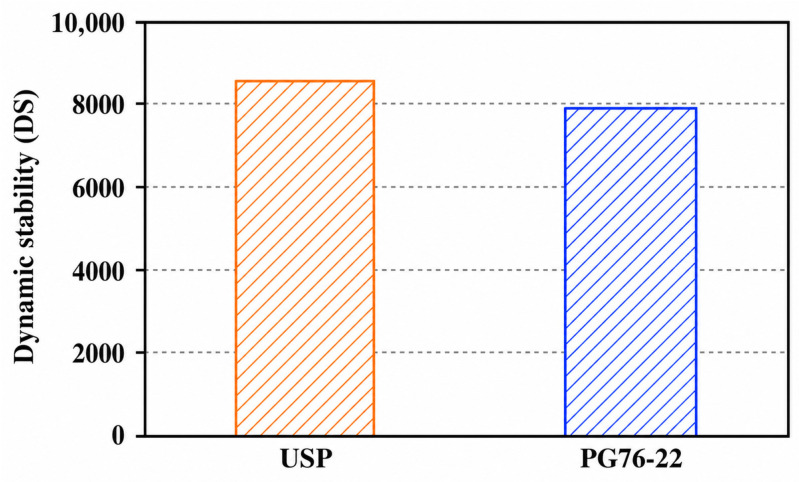
Rutting test results.

Figure 17 compares the rutting test results of the two asphalt mixtures. The dynamic stability of the USP-modified PG76-22 asphalt mixture is 6.2% higher than that of the PG76-22 asphalt mixture, indicating that the USP modifier does not weaken the high-temperature rutting resistance at the mixture scale. Its high-temperature stability is comparable to, and slightly better than, that of the PG76-22 asphalt mixture. This result is not completely consistent with the binder rheological tests, in which the rutting factor of the USP-modified asphalt binder decreases. This is because rheological tests mainly characterize the viscoelastic properties of the asphalt binder itself, while mixture rutting tests reflect the combined effects of asphalt binder, aggregate skeleton, air void structure, and interfacial adhesion.

From the perspective of mixture structure, the difference in the optimum asphalt–aggregate ratio provides a direct mixture-design explanation for the rutting results. The optimum asphalt–aggregate ratio of the USP-modified PG76-22 asphalt mixture was 4.2%, which was lower than that of the PG76-22 asphalt mixture (4.7%). A lower asphalt content can reduce the amount of free or movable asphalt binder in the mixture at high temperature, thereby limiting binder-related flow deformation under wheel loading. Under the same AC-20 gradation, this also means that the rutting response of the compacted mixture may depend more strongly on the aggregate skeleton and particle interlock.

In addition, the alkaline surface of limestone aggregate shows good interfacial compatibility with the polar components in the asphalt binder system [34], which may be beneficial to asphalt–aggregate bonding in the USP-modified mixture. However, because direct adhesion, coating, and interface strength tests were not conducted in this study, the contribution of interfacial bonding should be further verified in future work.

#### 3.4.2. Moisture Stability Analysis

**Figure 18:**
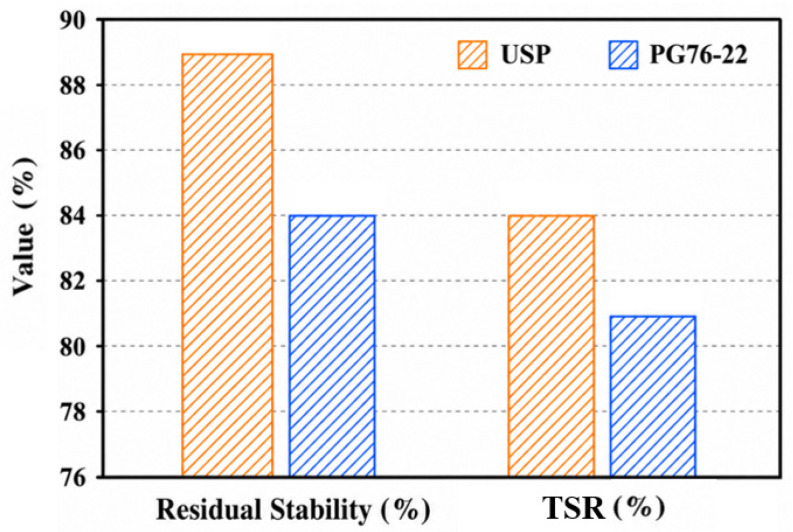
Comparison of moisture stability.

Figure 18 compares the moisture stability of the USP-modified PG76-22 asphalt mixture and the PG76-22 asphalt mixture. After adding the USP modifier, the residual stability of the mixture increases by approximately 5%, and the TSR increases by approximately 3%. This improvement may be related to better coating and interfacial adhesion between the asphalt binder and limestone aggregates after USP modification, which could reduce moisture-induced stripping under immersion and freeze–thaw conditioning. However, since direct interface characterization was not performed in this study, this explanation should be regarded as a potential mechanism that needs further verification.

Although the increases in residual stability and TSR were relatively small, approximately 5% and 3%, respectively, the coefficients of variation were low and the differences between the two mixtures were statistically significant (*p* < 0.05). This indicates that the improvement in moisture stability after USP modification was not caused by random test variability. The detailed statistical results are summarized in Section 3.5.

#### 3.4.3. Fatigue Performance Analysis

The four-point bending fatigue test results are shown in Table 11. At strain levels of 300, 400, and 500 με, the fatigue life of the USP-modified PG76-22 asphalt mixture is higher than that of the PG76-22 asphalt mixture. Specifically, the fatigue life increases by approximately 23.0%, 57.9%, and 40.5% at 300, 400, and 500 με, respectively. This indicates that the USP-modified PG76-22 asphalt mixture exhibited better fatigue resistance than the PG76-22 asphalt mixture under the tested strain-controlled four-point bending conditions. In addition, the average initial stiffness of the USP-modified PG76-22 asphalt mixture is 4652 MPa, lower than the 5782 MPa of the PG76-22 asphalt mixture, suggesting that the modified mixture has lower stiffness, improved flexibility, and better deformation coordination.

According to the statistical results in Table 12, the CV values of fatigue life under the three strain levels were all within an acceptable range, indicating that the fatigue test results were repeatable. The increases in fatigue life of the USP-modified mixture at 300, 400, and 500 με were statistically significant (*p* < 0.05), confirming the beneficial effect of the USP modifier on mixture-level fatigue performance.

**Figure 19:**
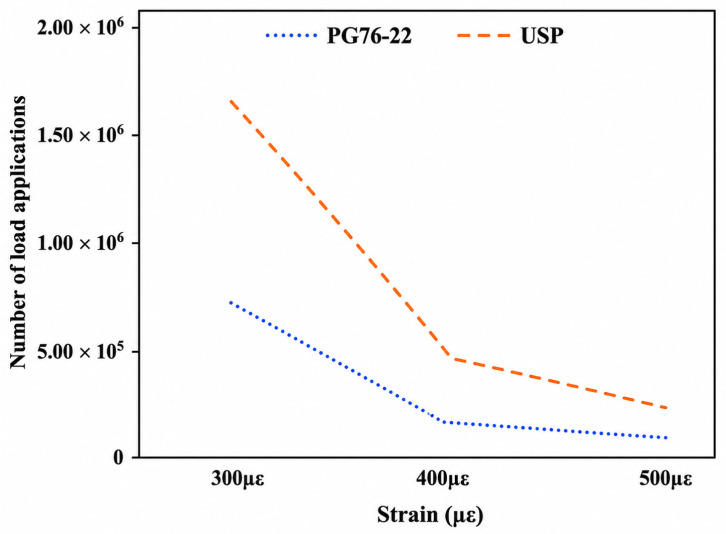
Comparison of number of load cycles.

Figure 19 compares the fatigue performance of the USP-modified asphalt mixture and the PG76-22 asphalt mixture. The results show that the USP-modified PG76-22 asphalt mixture exhibited longer fatigue life at all tested strain levels. This improvement should be interpreted from a mixture-scale perspective. The USP-modified mixture had a lower average initial stiffness than the PG76-22 mixture, indicating greater flexibility and better deformation coordination under cyclic bending. These characteristics may help to reduce stress concentration and delay crack propagation in the mixture. In addition, the warm-mix effect of the USP modifier may improve mixture workability and reduce thermal aging during preparation; however, this was not directly verified in the present study. Therefore, the improved four-point bending fatigue life should be attributed primarily to the measured mixture-scale response, while the possible contributions of workability, aging, and interfacial behavior require further investigation.

The different trends observed in the LAS and four-point bending fatigue tests indicate a scale-dependent fatigue response of asphalt materials. The LAS test mainly evaluates binder-scale fatigue damage under controlled shear loading, whereas the four-point bending fatigue test reflects mixture-scale cracking resistance under cyclic bending. Although USP modification reduced the binder fatigue life in the LAS test, the USP-modified mixture exhibited lower initial stiffness and longer fatigue life under the tested strain levels, suggesting improved flexibility and deformation coordination at the mixture scale. Therefore, binder-scale fatigue results cannot be directly extrapolated to mixture-scale performance, which may also be influenced by asphalt content, aggregate structure, air void characteristics, and asphalt–aggregate interaction. Further volumetric analysis, interface characterization, and aging comparison tests are needed to verify the underlying mechanisms.

### 3.5. Statistical Analysis

To further evaluate the dispersion and significance of the experimental results, the main performance indicators were statistically summarized, and the results are shown in Table 12.

For each performance test, parallel specimens or repeated measurements were prepared according to the corresponding test standards. The results are reported as mean values with standard deviations. The coefficient of variation (CV) was calculated to evaluate the repeatability of the test results. Since most comparisons in this study involved two material systems, namely the PG76-22 asphalt binder/mixture and USP-modified PG76-22 asphalt binder/mixture, independent-samples t-tests were used to evaluate statistical significance. A significance level of *p* < 0.05 was adopted.

Overall, the coefficients of variation for all indicators were relatively low, indicating good repeatability of the test results. The significance analysis showed that the effects of the USP modifier on the main performance indicators of the PG76-22 asphalt binder and its mixtures were statistically significant. These results further confirm that the influence of the USP modifier on material performance is statistically meaningful.

## 4. Conclusions

In this study, microscopic characterization tests, asphalt binder performance tests, and asphalt mixture performance tests were conducted to investigate the effects of the USP modifier on the PG76-22 asphalt binder and its mixtures. The main conclusions are as follows:

(1) The FM results indicate that the USP modifier can be relatively uniformly dispersed in the PG76-22 asphalt binder under the adopted preparation conditions. The FTIR results show that no distinct new absorption peaks appeared after USP modification, and the spectral changes were mainly reflected in the relative intensities and peak area ratios of existing characteristic absorption bands. The DSC results show that the USP modifier exhibits an endothermic transition in the range of 88–99 °C, which is related to its thermal softening behavior.
(2) The USP modifier enhances the low-temperature performance of the PG76-22 asphalt binder. With the addition of USP, the ductility of the PG76-22 asphalt binder at 5 °C increases by 66.7%. This significant increase indicates improved low-temperature ductility and resistance to cracking.
(3) The USP modifier reduces the high-temperature deformation resistance of the PG76-22 asphalt binder at the binder scale. The PG grading, MSCR, and temperature sweep results show that USP modification lowers the rutting factor, increases non-recoverable deformation under high stress, and enhances stress sensitivity.
(4) The influence of the USP modifier on fatigue performance was scale-dependent. The LAS results showed that the USP-modified asphalt binder had a shorter estimated fatigue life than the PG76-22 asphalt binder, whereas the four-point bending fatigue test showed that the USP-modified asphalt mixture had a longer fatigue life under the tested strain levels. This difference indicates that binder-scale fatigue indices cannot be directly extrapolated to mixture-scale fatigue performance.
(5) The USP-modified PG76-22 asphalt mixture showed slightly improved mixture-level performance under the present laboratory conditions. Its dynamic stability increased by 6.2%, while the residual stability and freeze–thaw splitting tensile strength ratio increased by approximately 5% and 3%, respectively.

However, challenges remain in terms of dosage optimization, aging characterization, volumetric analysis, and the quantitative correlation between binder-scale rheological properties and mixture-scale pavement performance. Future work will focus on evaluating different USP contents, characterizing aging resistance and volumetric properties, and establishing quantitative relationships among modifier dispersion morphology, binder rheology, and mixture performance.

## Figures and Tables

**Table 1:** Main properties of PG76-22 asphalt binder.

Test	Unit	Technical Requirement	Test Result
Penetration (25 °C, 100 g, 5 s)	0.1 mm	≥40	64
Softening point	°C	≥80	92.5
Ductility (5 °C)	cm	≥20	36
Kinematic viscosity (135 °C)	Pa·s	≤3.0	2.85

**Table 2:** Main properties of the USP modifier.

Test	Unit	Technical Requirement	Test Result
Appearance	/	Black	Black or purple-black paste
Odor	/	No obvious odor	No obvious odor
Flash point	°C	≥145	210
Density	g/cm^3^	0.94–0.99	0.985
Water content	%	<0.1	0.05
Ash content	%	<0.15	0.1
Solubility at 20 °C	%	≥99.5	99.9
Rotational viscosity at 135 °C	Pa·s	0.010–0.014	0.012
pH value	/	Neutral	Neutral

**Table 3:** Basic properties of coarse aggregate.

Property	Test Result	Specification Requirement	Test Method
Crushing value (%)	21	≤26	T 0316
Los Angeles abrasion value (%)	16.8	≤28	T 0317
Water absorption (%)	1.5	≤2.0	T 0304
Soundness (%)	3.5	≤12	T 0314
Soft particle content (%)	1.2	≤3.0	T 0320
Flat and elongated particle content (%)	10.5	≤15	T 0312

**Table 4:** Basic properties of fine aggregate.

Property	Test Result	Specification Requirement	Test Method
Clay content (%)	2.1	≤3	T 0333
Apparent relative density	2.635	≥2.50	T 0304
Sand equivalent (%)	76.7	≥60	T 0334
Soundness (%)	4.2	≤12	T 0340

**Table 5:** Basic properties of limestone mineral filler.

Property	Test Result	Specification Requirement	Test Method
Density (g·cm^−3^)	2.68	>2.5	T 0352
Hydrophilic coefficient	0.60	<1	T 0353
Water content (%)	0.18	<1	T 0103
BET specific surface area (m^2^·g^−1^)	0.49	—	—

**Table 6:** Gradation design of the AC-20 asphalt mixture.

Gradation	26.5	19.0	16.0	13.2	9.5	4.8	2.4	1.2	0.6	0.3	0.2	0.1
Upper limit	100.0	100.0	92.0	80.0	72.0	56.0	44.0	33.0	24.0	17.0	13.0	7.0
Lower limit	100.0	90.0	78.0	62.0	50.0	26.0	16.0	12.0	8.0	5.0	4.0	3.0
Median gradation	100.0	95.0	85.0	71.0	61.0	41.0	30.0	22.5	16.0	11.0	8.5	5.0
Designed combined gradation	100.0	93.7	83.4	73.0	61.8	38.7	27.3	18.5	15.5	11.4	8.5	7.0

**Table 7:** Image processing results of fluorescence microscopy images.

Binder Type	Average Equivalent Particle Diameter (μm)	Particle Area Fraction (%)	Number Density (Particles·mm^−2^)	Dispersion Coefficient
USP-modified PG76-22 asphalt binder	6.08	0.20	57.66	0.45
PG76-22 asphalt binder	—	—	—	—

**Table 8:** Quantitative FTIR indices of different asphalt binders.

Binder Type	Carbonyl Index	Sulfoxide Index	A2920/A1456	A1456/A1374
PG76-22 asphalt binder	0.026	0.010	1.53	1.97
USP-modified PG76-22 asphalt binder	0.029	0.018	2.83	2.05

The A2920/A1456 and A1456/A1374 ratios were used to characterize the relative variation in aliphatic C–H structures after USP modification.

**Table 9:** Effect of the USP modifier on the properties of the PG76-22 asphalt binder.

Asphalt Binder Type	Failure Temperature (°C)	Rutting Factor (kPa)	Fatigue Factor (kPa)
USP-modified asphalt binder	78.9	1.27	1.08
USP asphalt binder after RTFOT	78.19	2.66	2.04
PG76-22 asphalt binder	81.46	1.61	1.38

**Table 10:** Fatigue performance parameters of asphalt binders.

Material	a	*C*_1_	*C*_2_	A	B	*N_f_*	
						*N_f_* at 2.5% Strain	*N_f_* at 5% Strain
USP	2.327	0.128	0.387	1.31 × 10^7^	4.654	1.84 × 10^5^	7.32 × 10^3^
PG76-22	2.339	0.189	0.378	2.97 × 10^7^	4.667	4.13 × 10^5^	1.62 × 10^4^

**Table 11:** Number of load cycles.

Strain Level	300 με	400 με	500 με	Average Initial Stiffness (MPa)
PG76-22	725,650	125,784	40,957	5782
USP-modified	892,475	198,620	57,560	4652

**Table 12:** Statistical summary of main performance indicators.

Indicator	PG76-22	USP-Modified	CV Range (%)	*p*-Value
Penetration (0.1 mm)	64.0 ± 1.2	50.0 ± 0.9	1.8–1.9	<0.05
Softening point (°C)	92.5 ± 0.8	84.0 ± 0.7	0.8–0.9	<0.05
Ductility at 5 °C (cm)	36.0 ± 1.5	60.0 ± 2.0	3.3–4.2	<0.05
Failure temperature (°C)	81.46 ± 0.42	78.90 ± 0.38	0.5–0.6	<0.05
Rutting factor (kPa)	1.61 ± 0.04	1.27 ± 0.03	2.4–2.5	<0.05
Fatigue factor (kPa)	1.38 ± 0.04	1.08 ± 0.03	2.8–2.9	<0.05
R_0.1_ (%)	86.0 ± 1.6	92.0 ± 1.8	1.9–2.0	<0.05
R_3.2_ (%)	47.0 ± 1.2	44.0 ± 1.3	2.6–3.0	<0.05
$J_{nr,0.1}$	58.0 ± 2.3	40.0 ± 1.8	4.0–4.5	<0.05
$J_{nr,3.2}$	265 ± 12	350 ± 16	4.5–4.6	<0.05
$J_{nr,diff}$	205 ± 10	310 ± 14	4.5–4.9	<0.05
Dynamic stability (passes·mm^−1^)	7900 ± 320	8400 ± 350	4.1–4.2	<0.05
Residual stability (%)	84.8 ± 1.5	89.0 ± 1.6	1.8–1.9	<0.05
TSR (%)	81.6 ± 1.3	84.0 ± 1.4	1.6–1.7	<0.05
Fatigue life at 300 με	725,650 ± 31,200	892,475 ± 38,600	4.3	<0.05
Fatigue life at 400 με	125,784 ± 5680	198,620 ± 8740	4.4–4.5	<0.05
Fatigue life at 500 με	40,957 ± 1960	57,560 ± 2640	4.6–4.8	<0.05
Initial stiffness (MPa)	5782 ± 218	4652 ± 186	3.8–4.0	<0.05

## Data Availability

The original contributions presented in this study are included in the article. Further inquiries can be directed to the corresponding author.

## Abbreviations

The following abbreviations are used in this manuscript:

USP	Usual-Temperature Synthetic Pitch
PG76-22	Performance Grade 76-22
AC	Asphalt Concrete
RTFOT	Rolling Thin Film Oven Test
